# Fabrication of superconducting niobium nitride nanowire with high aspect ratio for X-ray photon detection

**DOI:** 10.1038/s41598-020-65901-5

**Published:** 2020-06-03

**Authors:** Shuya Guo, Qi Chen, Danfeng Pan, Yaojun Wu, Xuecou Tu, Guanglong He, Hang Han, Feiyan Li, Xiaoqing Jia, Qingyuan Zhao, Hengbin Zhang, Xiaomin Bei, Jun Xie, Labao Zhang, Jian Chen, Lin Kang, Peiheng Wu

**Affiliations:** 1 Research Institute of Superconductor Electronics, Nanjing University, Hankou Road 22, Nanjing, 210093 China; 2 Qian Xuesen Laboratory of Space Technology, Beijing, 100094 China

**Keywords:** Astronomy and planetary science, Engineering, Materials science, Astronomy and astrophysics

## Abstract

The niobium nitride (NbN) nanowires fabricated with the high-quality ultra-thin NbN film with a thickness of 3 nm–6 nm were widely used for single photon detectors. These nanowires had a low aspect ratio, less than 1:20. However, increasing the thickness and the aspect ratio of highly-uniformed NbN nanowires without reducing the superconductivity is crucial for the device in detecting high-energy photons. In this paper, a high-quality superconducting nanowire with aspect ratio of 1:1 was fabricated with optimized process, which produced a superconducting critical current of 550 μA and a hysteresis of 36 μA at 2.2 K. With the optimization of the electron beam lithography process of AR-P6200.13 and the adjustion of the chamber pressure, the discharge power, as well as the auxiliary gas in the process of reactive ion etching (RIE), the meandered NbN nanowire structure with the minimum width of 80 nm, the duty cycle of 1:1 and the depth of 100 nm were finally obtained on the silicon nitride substrate. Simultaneously, the sidewall of nanowire was vertical and smooth, and the corresponding depth-width ratio was more than 1:1. The fabricated NbN nanowire will be applied to the detection of soft X-ray photon emitted from pulsars with a sub-10 ps time resolution.

## Introduction

Niobium nitride (NbN) has been widely studied in superconductor electronics for high superconducting transition temperature (up to 16 K), critical current density and critical magnetic field^1^. Moreover, the corresponding physical properties were stable, and it could have large scale with high quality. Also, it was the most common materials to fabricate the superconducting nanowire single-photon detectors (SNSPDs)^2,3^, the mixer of Terahertz wave detection hot-electron-bolometer (HEB)^4^, and the superconducting quantum interference device (SQUID). At present, the preparation of SNSPDs mainly use the epitaxial method to grow the high-quality ultra-thin NbN film with a thickness of 3–6 nm. Then, the film is prepared to the meandered nanowire structure by means of micro-nano processing. The width of NbN nanowire is about 100 nm, and the aspect ratio is generally lower than 1:20.

Considering the advantages of SNSPDs, such as the low dark count, the wide response spectrum, the short recovery time and the high time precision, SNSPDs were widely studied and applied to the detection of visible/infrared single photon^5^. However, the application of SNSPDs in the direction of high-energy photon (UV, X-ray, and gamma ray) was rarely studied for low absorption of ultra-thin NbN films with the high-energy photons^6–8^. Through Geant4 simulations, we obtained that the X-ray photon absorption of the 10 nm thick NbN film for 1 keV and 6 keV was only 3.69% and 0.23%, respectively^9^, and the SNSPD prepared with the ultra-thin NbN film was unable to effectively detect high-energy single photons. The simulation results show that the absorption of 1 keV and 6 keV by NbN with a 100 nm film thickness are 31.31% and 2.25%, respectively. To improve the absorption of superconducting NbN nanowire detector in the high-energy photon field, the development of superconducting NbN nanowire with high aspect ratio is the key component.

In this paper, high-quality superconducting nanowires with a thickness of 100 nm and an aspect ratio of 1:1 were developed by introducing a new anti-etching agent called AR-P 6200.13. The fabricated nanowire produced a superconducting critical current of 550 μA and a hysteresis of 36 μA at 2.2 K. The NbN nanowire may be applied to the soft X-ray photon detection with high time resolution.

## Experiments

In the experiment, the electron beam lithography technology (EBPG 5200) with the highest accuracy of 8 nm was utilized to obtain the nanowire image. Speaking of deep etching, the well-known Bosch process in deep reactive ion etching (DRIE) technology is widely used in high aspect ratio silicon etching. However, the time-multiplexed alternating process by SF_6_ etching and CF_x_ polymerization steps would form the ripples on the sidewall^9^ and result in large roughness. Although the optimized DRIE process can reduce the sidewall roughness to approximately 10 nm^10^, it is still too rough for NbN nanowires with only 100 nm width. Considering the simple reactive ion etching (RIE) process continuously performs gas conversion and the sidewall of the nanowire is much smoother, it is more suitable for the demands of high sensitivity detection. Besides, the anisotropic etching can be guaranteed when the etching depth reaches the order of 100 nm, which is the main method for NbN etching^2,4,5,11^. The challenge is that all chemical reactions in the RIE process lead to the sideways etching and enhance the difficulty of etching thick NbN film. This problem can be effectively addressed by optimizing the etching parameters. Therefore, RIE etching is selected to prepare NbN nanowires with a high aspect ratio.

In the experiment, NbN nanowire with the thickness of 100 nm, the minimum width of 80 nm, the aspect ratio of over 1:1 and the vertical as well as smooth sidewall is obtained by optimizing the parameters of electron beam lithography and reactive ion etching. At the same time, the preparation of NbN nanowires with a 200 nm film thickness is also further explored, providing a technological basis for the later development of superconducting NbN nanowires high-energy photon detectors.

### Fabrication of NbN nanowires

**Figure 1:**
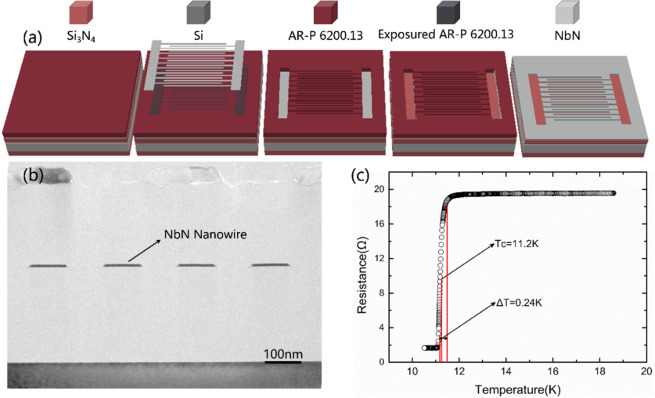
The preparation process and the characterization of NbN nanowire. (**a**) The schematic diagram of preparation process for NbN nanowire with high aspect ratio: Spining coating, Elctron-beam lithography, Development, Reactive ion etching, Degumming; (**b**) TEM image of ultra-thin NbN nanowire with the aspect ratio of about 1:20; (**c**) Superconducting transition temperature(Tc) is 11.2 K for NbN film with thickness of 100 nm, which grows on Silicon substrate with a Si_3_N_4_ layer, and the superconducting transition width(ΔT) is 0.24 K.

How to accurately prepare electron beam lithography (EBL) pattern and how to transfer EBL pattern to NbN film are the essential problems that should be taking into consideration. Figure 1(a) shows the process flow diagram of superconducting NbN nanowire with high aspect ratio^12^. Firstly, NbN film grows on the Si_3_N_4_/Si/Si_3_N_4_ substrate, which polishs with double sided. Then, AR-P 6200.13 (a positive electron-beam resist) is spin-coated on the surface of NbN film. Moreover, the homogenizer speed is 4000 r/min, the spin-coating time is 60 s, and the thickness of resist is about 400 nm. In addition, the resist is baked for 60 s with the temperature of 150 °C to remove the organic solvent. The model of electron beam lithography machine is EBPG5200 of Germany Raith company, and the electron beam flow is generally 0.1–1 nA with the exposure dose adjusted according to pattern size. After the exposure, AR 600-546 is used to develop at 20 °C, the deionized water is used to fix for 60 s, and the glue is used to firm at the temperature of 130 °C for 60 s to enhance the corrosion resistance. After electron beam lithography and development, RIE (SAMCO International) is used to transfer the exposure pattern to NbN film. The ranges of parameters in experiment as following: the discharge power is 50 W–100 W, the etching gas is CF_4_, the gas flow is 30 sccm, the chamber pressure is 1–4 Pa. Finally, AR 600-71 is used to remove the residual resist, and the low-power ultrasound can be used to remove the resist. The results of exposure and etching morphology are observed by the scanning electron microscope of MERLIN Compact from Zeiss. Meanwhile, the cross-section of nanowire is analyzed by means of transmission electron microscope. The TEM image is shown in Fig. 1(b). It can be seen that the aspect ratio of nanowire is just about 1:20, which can not be applied to the high-energy photon detection.

### Fabrication of NbN films

Niobium nitride (NbN) film is the most commonly used superconducting material for SNSPDs. In addition to NbN, Nb, NbTiN^13^, TaN^14^, WSi^15^, MoSi^16^ are also commonly used to prepare SNSPDs. The model of magnetron sputtering system used in experiment is DE500 of Deyi Tech. company. Moreover, NbN film was grown on Si3N4/Si/Si3N4 substrate based on DC magnetron sputtering with the sputtering rate of 1.25 nm/s. Also, the sputtering times two types of NbN thin film were grown over 80 s and 160 s respectively, and the thickness are 100 nm and 200 nm. The square resistance of NbN film with thickness of 100 nm is 23.6 Ω, which is measured by four-probe method at room temperature. The superconducting transition temperature (Tc) and the superconducting transition width (ΔT) have measured values of 11.2 K and 0.24 K, respectively. Here, Tc is defined at 10%R (R is the resistance of the normal state at 20 K), and ΔT is the temperature interval between 90%R and 10%R^17^. Meanwhile, the test results are shown in Fig. 1(c), and it is shown that the film has good superconducting characteristics which can be used for the subsequent preparation of superconducting nanowire high-energy single photon detector.

### Optimization of the AR-P 6200.13 electron beam exposure process

The EBPG 5200 with the highest electron acceleration voltage of 100 kV is a Gaussian beam vector scanning exposure system. In the process of electron beam exposure, the parameters such as resolution, scanning times, writing field size, beam flow and exposure dose are generally optimized to improve the exposure quality. In the experiments, the step size of lithography is set to 1 nm to improve the exposure accuracy. Since the exposure area of nanowire is relatively small, the writing field size is generally set to 340 μm × 340 μm, so as to reduce the splicing error between different writing fields. To make the nanowire edge flat, the scanning times are generally 4, and the exposure dose is adjusted to 1/4 of the original value. According to the repeated scanning, the edge flatness of nanowire can be further improved by the repeated scanning. The following equation can be met in the process of electron beam lithography: Exposure time × Electron beam flow = Exposure dose per unit area × Exposure area.

As for the electron beam lithography system, the exposure accuracy decreases with the spot size of electron beam increases. In the process of experiment, smalller size is generally used for exposure, and the beam is generally set to 100 pA for the high-precision pattern. As for the large-scale pattern with low accuracy requirement, the beam with more than 1 nA is generally used for exposure. Also, the exposure time is controlled within two hours by adjusting different beam to improve the stability of exposure accuracy.

AR-P 6200.13 is a non-chemical amplification positive electron beam resist with a maximum resolution of less than 10 nm, high sensitivity, and fast exposure speed. Meanwhile, its excellent dry etching resistance is two times that of the traditional PMMA adhesive, which can be used to replace ZEP520^18^. The high resolution and etching resistance of AR-P 6200.13 are suitable for making high aspect ratio nanowires, which are the key factors for the success of our experiments. Due to the use of Si3N4 substrate and the thickness of AR-P 6200.13 resist up to 400 nm, the charge accumulation and scattering during electron beam lithography can strengthen the proximity effect^19^. To get the ideal line width, NbN nanowire structure with uniform line width needs to strictly optimize the conditions of electron beam lithography.

In this study, the wandering nanowire with different line widths and duty cycle of 1:1 was designed to conduct different dose exposure experiments. There is some deviation between the designed line width and the actual line width due to the proximity effect. On account of the same exposure dose, the deviation increases with the line width increases. As the line width increases, the exposure area increases, and the total number of exposure electrons increases, which leads to the serious overexposure. As for designing the graphics, it is necessary to adjust the line width/spacing (L/S) of target graphics according to the exposure condition, and to compensate the line width with different graphics sizes^20^, so as to correct the proximity effect. Moreover, Table 1 shows the actual line width of various nanowires measured by SEM under the conditions of different exposure doses.

**Table 1:** The measured line widths of nanowires with different exposure doses.

Exposure Width(nm)	Designed Width(nm)	Exposure Dose (μC/cm^2^)				
		80	85	90	95	100
60	L/S = 100/20	Χ	Χ	Χ	35/85	Χ
80	L/S = 120/40	Χ	88/72	76/84	80/80	68/92
100	L/S = 150/50	110/90	105/95	102/98	101/97	84/116
120	L/S = 170/70	131/109	125/115	116/124	106/134	91/149
150	L/S = 210/90	173/127	162/138	155/145	146/154	133/167

**Figure 2:**
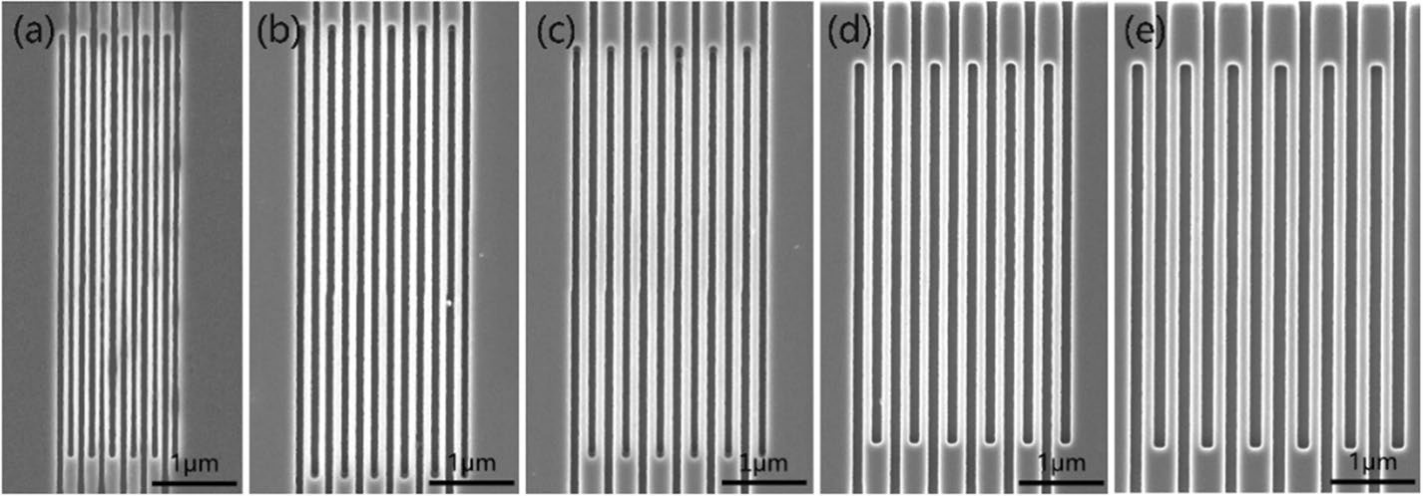
The SEM images of NbN nanowires with different line widths. (**a**) L/S = 60 nm/60 nm, (**b**) L/S = 80 nm/80 nm, (**c**) L/S = 100 nm/100 nm, (**d**) L/S = 120 nm/120 nm, (**e**) L/S = 150 nm/150 nm.

According to the measurement data in Table 1, it can be seen that the exposure window is much narrow for the exposure of pattern below 80 nm, and the image is difficult to be prepared. Meanwhile, the exposure dose should not be too small, otherwise the exposure amount is insufficient, which can result in underexposure. Moreover, the resist can not play fully during development, which can result in the resist adhesion. However, if the exposure dose is slightly large, the resist line becomes much narrow after development, which deviates from the design. In addition, the resist line is easy to drift and collapse. As the design target line width/interval (L/S) is 80 nm/80 nm, it is necessary to adjust L/S of design pattern to 120/40. Then, 4 scanning exposures are conducted, the exposure dose can only be in the range of 90-95 μC/cm^2^. Figure 2(a–e) show SEM images of nanowire with the line width of 60 nm, 80 nm, 100 nm, 120 nm and 150 nm after exposure etching as the exposure dose is 92 μC/cm^2^. It can be seen that the line gap of nanowire with L/S of 60 nm/60 nm is uneven, the proximity effect is too large, and the resist line after exposure development is too thin, so the adhesion with substrate is weakened, and the etching performance is weakened. Therefore, the resist correspondingly drifts and collapses, while other nanowires in Fig. 2(b–e) have uniform width, which is consistent with the experimental design. If it is supposed to get the nanowire lower than 60 nm, it is necessary to use thin electron beam resist to improve the process resolution by reducing the proximity effect.

### Optimization of the RIE etching process

To transfer the meandered nanowire pattern to NbN film and obtain the structure with high aspect ratio, Reactive Ion Etching (RIE) technology is used in this study. RIE process is much complex as involving both physical and chemical process, which has multiple adjustable parameters, such as gas flow, discharge power, chamber pressure, and density as well as distribution of etching pattern. Meanwhile, each parameter can affect the final etching results^21^.

During the experiment, NbN films were etched by the mixture gas (SF_6_, CF_4_, CHF_3_). Also, O_2_, Ar, H_2_ and other gases can be added to control the etching rate, the etching direction and the anti-etching ratio of mask. Also, many groups use CF_4_ gas to etch NbN^22–24^, and it is verified that CF_4_ gas can obtain vertical and steep nanowire groove^23^. Therefore, CF_4_ gas is selected to etch NbN film in the experiment, and the reaction chemical equation is shown as following^25,26^:

$${{\rm{CF}}}_{4}+2{\rm{NbN}}\to 2{{\rm{NbF}}}_{3}\uparrow +{{\rm{N}}}_{2}\uparrow +2{\rm{CF}}\uparrow $$

Firstly, we set the gas flow parameter value to 30 sccm referencing the instructions of equipment (Samco RIE-10NR) and our previous work^2^. Then, the discharge power and the chamber were optimized as follows.

**Figure 3:**
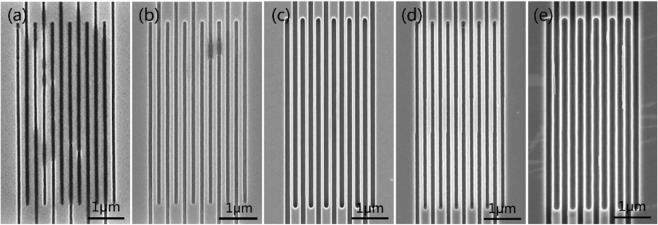
SEM images of an L/S with 100 nm/100 nm nanowires prepared by different etching recipes. (**a**) Recipe 1 etching results; (**b**) Recipe 2 etching results; (**c**) Recipe 3 etching results; (**d**) Recipe 4 etching results; (**e**) Recipe 5 etching results.

Table 2 shows the etching process recipe and Fig. 3 shows the SEM image of the L/S with 100 nm/100 nm nanowires etched by the corresponding recipe. We set the gas pressure parameter lower in recipes 1-3. This is because the lower gas pressure reduces the collision between ions, ions and atoms, and the lateral etching of the material by ion bombardment is reduced, thereby improving the anisotropy of the etching^27^. The discharge power is set to 50 W, 80 W, and 100 W. Figure 3(a–c) show the etching effect diagrams. It can be seen from Fig. 3(a) that when the discharge power is 50 W, there is a large amount of adhesion on the surface of the nanowires, indicating the film is not completely engraved. When the etching power is 80 W, there is a small amount of adhesion on the surface of the nanowires and most places have been engraved. The nanowire line width is approximately 110 nm, which does not meet the 100 nm line width requirement. When the etching power is set to 100 W, it can be clearly seen from Fig. 3(c) that the nanowire width is less than 100 nm. This indicates the power is too high, which causes the nanowire to etch severely in the lateral direction.

**Table 2:** The RIE process recipes.

Recipe	Etching Gas	Discharge Power	Gas Flow	Chamber Pressure
1	CF_4_	50 W	30sccm	1.2 Pa
2	CF_4_	80 W	30sccm	1.2 Pa
3	CF_4_	100 W	30sccm	1.2 Pa
4	CF_4_	80 W	30sccm	2 Pa
5	CF_4_	80 W	30sccm	4 Pa

In the first three sets of experiments, the gas pressure parameters were set low, which resulted in film that was not completely engraved and the lines were too wide when the power was low. In the next two sets of experiments, we increased the gas pressure in the chamber to 2 Pa and 4 Pa, respectively. At the same time, the etching power setting is optimized to 80 W. As can be seen from Fig. 3(d,e), when the chamber pressure is 2 Pa, the nanowire width is 100 nm, which is the ideal width. When the gas pressure is 4 Pa, the nanowire width is significantly less than 100 nm, which indicates the gas pressure is too high, causing serious lateral etching^27^. After several groups of experimental comparisons were made, we chose etching recipe 4 which produced a width approaching our design.

**Figure 4:**
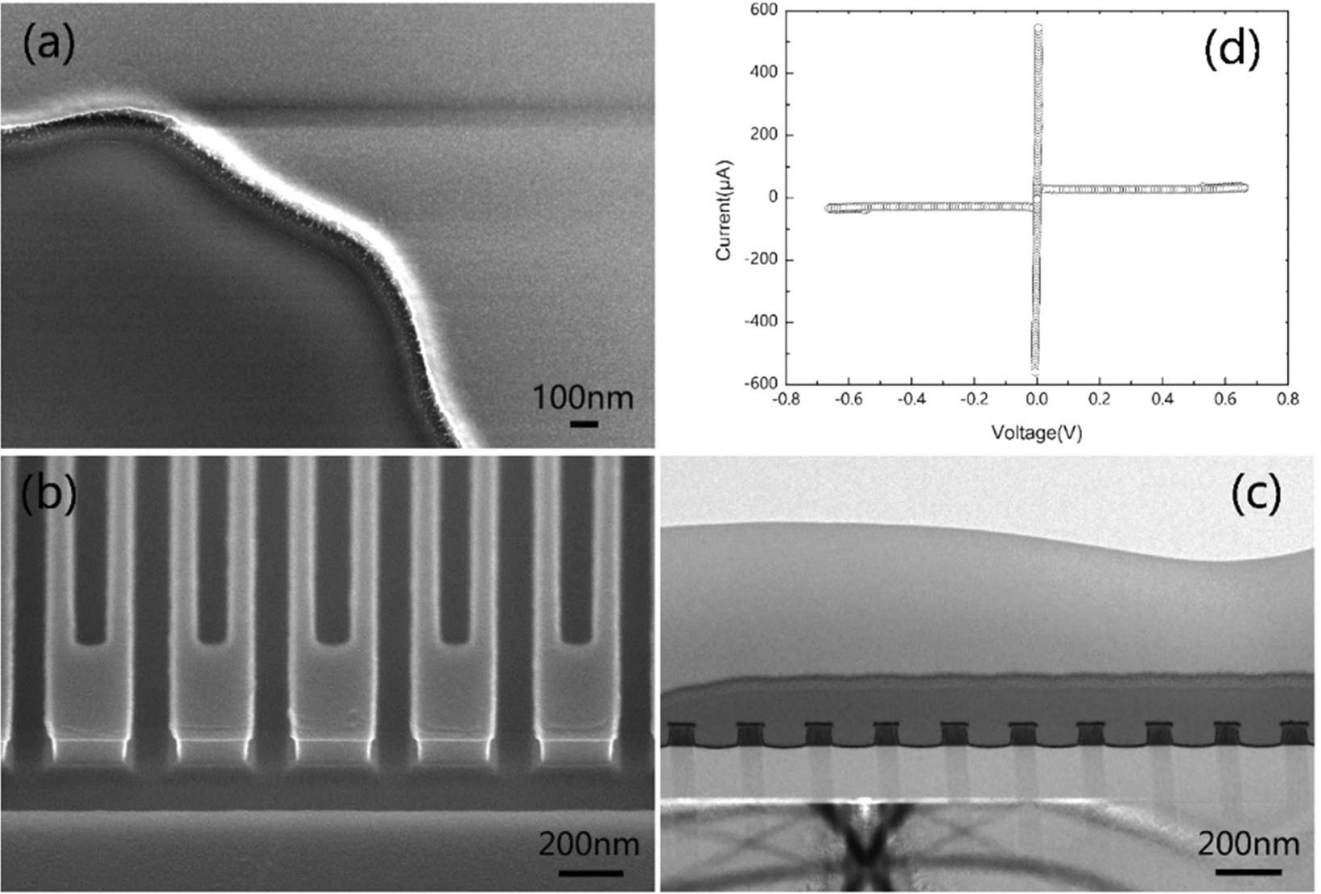
The morphology and electrical properties of NbN nanowire with a high aspect ratio. (**b**) SEM image of the side of the nanowire with an L/S of 80 nm/80 nm; (**c**) TEM image of the cross-section of the nanowire with an L/S of 80 nm/80 nm; (**d**) I-V curve of superconducting NbN nanowire with L/S of 80 nm/80 nm, the superconducting critical current Ic of 550 μA and the hysteresis of 36 μA.

Before the etching process of NbN nanowire, NbN film sample is generally selected to estimate the etching time, which is plated with electrode. By observing the sample surface color after etching, we can judge whether the film is fully etched. As the film sample is fully etched, it will present the dark red color of Si substrate with Si_3_N_4_ layer. When etching recipe 4 was used to etch the NbN film for 2.5 min, the sample was completely etched, and the NbN etching rate achieved was 40 nm/min. Figure 4(a) is the side SEM image of NbN film. Also, it is shown that the etching surface has good verticality. Since the groove gap of nanowire is narrow, the gas exchange rate is low, the etching rate is relatively slow, and the lag effect of RIE is serious^28,29^. Thus, it is necessary to extend the etching time of NbN nanowire by about 30 s to ensure that the nanowire can be completely etched. In the experiment, the resistance at both ends of nanowire is also measured to determine whether the film is fully etched. At the room temperature, the square resistance of NbN film with the thickness of 100 nm is 23.6 Ω, and the total length of nanowire is 60 μm. The total resistance of nanowire with width of 80 nm is about 17.7 kΩ. According to RIE recipe 4 in Table 2, the etching time is 3 min, and the measured resistance of nanowire with width of 80 nm is close to the theoretical resistance. Thus, it can be determined that NbN film with width of 100 nm has been completely etched and the nanowire has been prepared.

Figure 4(b,c) shows the side SEM of nanowire with L/S of 80 nm/80 nm and cross-section TEM image. It can be seen that the edge of nanowire etching is 90 degrees, and the side wall is smooth as well as flat. Meanwhile, there is a certain degree of over etching, and the maximum aspect ratio is more than 1:1. The current-voltage (I-V) characteristics of nanowire with L/S of 80 nm/80 nm are characterized by GM refrigerator at 2.2 K. Figure 4(d) shows the measured I-V curve. The measured superconducting critical current is 550 μA and the hysteresis is 36 μA, which indicates that the device has good performance and may be used in the high-energy single photon detection experiment.

### The preparation process research of NbN nanowire with thickness of 200 nm

**Figure 5:**
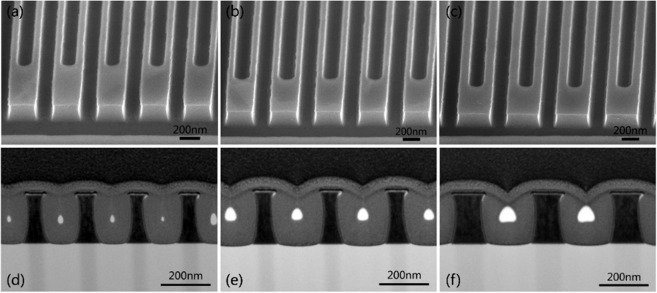
The SEM and TEM morphology of high aspect ratio nanowire with a thickness of 200 nm. (**a**) L/S of 100 nm/100 nm nanowire SEM image; (**b**) L/S of 120 nm/120 nm nanowire SEM image; (**c**) L/S of 150 nm/150 nm nanowire SEM image; (**d**) L/S of 100 nm/100 nm nanowire TEM image; (**e**) L/S of 120 nm/120 nm nanowire TEM image; (**f**) L/S of 150 nm/150 nm nanowire TEM image.

To further improve the absorption rate of NbN nanowire to X-ray photons, we try to use the film with a thickness of 200 nm to prepare the nanowire with high aspect ratio. The process flow is the same as above, and the etching time is extended to 6.0 min. Figure 5(a–c) shows the SEM images of designed nanowire with the line width of 100 nm, 120 nm and 150 nm, and Fig. 5(d–f) shows their corresponding TEM images of the cross-section of the nanowire after etching. Figure 5 shows that the width of the fabricated nanowires is uniform, but the lateral etching is more apparent. As shown in Fig. 5(a), for the L/S of 100 nm/100 nm nanowire, an L/S was prepared as 66 nm/134 nm. Due to the serious lateral etching, the line width was reduced by 30 nm. The duty cycle is changed to 2:1, and the aspect ratio reaches 3: 1. As shown in Fig. 5(b,c), the similar situation occurs for nanowires with L/S of 120 nm/120 nm and L/S of 150 nm/150 nm. At the same time, it can be clearly seen from the TEM image of Fig. 5(d–f) that the nanowires have a non-uniform line width in the vertical direction, with a narrow middle and wide ends. This is because the isotropy of the etching becomes more obvious with the extension of the etching time.

The above analysis shows that the physical bombardment effect of high-density plasma cannot be ignored when the AR-P 6200.13 electron beam resist is used as mask. Although the optimized RIE process conditions can reduce the lateral etching and isotropic etching, they cannot be completely eliminated. To etch the deep groove, the etching time needs longer time, and the transverse etching can result in the obvious line broadening compared with the line width of tens of nanometers. Thus, it is difficult to completely transfer the meandered nanowire structure with the duty cycle of 1:1 and the low L/S of 100 nm/100 nm to NbN film. To obtain the pattern of NbN nanowire with small period and high aspect ratio, it is necessary to use more etching resistant material as mask material (such as metal, silicon dioxide, etc.). Moreover, the pattern is firstly transferred to the mask material through lift-off process, and the material is used as mask for deep etching. Nowadays, the process in this field is still under study.

## Conclusions

According to the study of micro-fabrication technology, which combines the electron beam lithography system and RIE, the positive electron beam resist AR-P 6200.13 is used to form a complete preparation process of superconducting nanowire with high aspect ratio. Under the optimized conditions, the meandered NbN nanowire with L/S of 80 nm/80 nm is obtained with the thickness of 100 nm. The aspect ratio is more than 1:1, which can indicate the superconducting electrical properties of NbN nanowire with high aspect ratio. The preparation process of nanowire with high aspect ratio and film thickness of 200 nm is explored. Finally, the development of these technologies is prospective to the fabrication of SNSPD for high-energy single photon detection.
